# Personality of outpatients with malignant tumors: a cross-sectional study

**DOI:** 10.1186/1477-7819-10-187

**Published:** 2012-09-11

**Authors:** Zhuo Wang, Toshihiko Sakakibara, Yuichi Kasai

**Affiliations:** 1 Department of Spinal Surgery and Medical Engineering, Mie University Graduate School of Medicine, 2-174 Edobashi, Tsu city, Mie, 514-8507, Japan

**Keywords:** Cancer, Personality, Extroversion, Neuroticism, Psychology

## Abstract

**Background:**

There have been scarce large-scale studies investigating the personality of patients with malignant tumors. The purpose of this study is to determine the characteristic personality in malignant tumors outpatients.

**Methods:**

Three thousand and three among 5013 consecutive outpatients who consented to answer the Japanese Maudsley Personality Inventory questionnaires were divided into two groups. 603 outpatients diagnosed with malignant tumors (M group) and the other 2400 outpatients (non-M group) were enrolled in this study. We determined three scores such as introversion/extroversion (E-score), neuroticism (N-score), and lie detection (L-score). All data were used to compare the two groups.

**Results:**

Average E-score was slightly higher, and average N-score was slightly lower in M group than that in non-M group, and no significant differences between the two groups. However, the average L-score in M group was significant higher than that in non-M group (p < 0.01).

**Conclusion:**

Outpatients with malignant tumors showed a significantly higher L-score on MPI when compared with patients with non-malignant tumors. These results stress the importance of taking the mentality of patients with cancer into consideration when conducting treatment and care.

## Background

It has been suggested that the personality of patients with malignant tumors may have an effect on tumor development or progression [1-3], and although various studies on this topic have been conducted, large-scale studies have been scarce. Therefore, in this study we conducted a personality inventory in approximately 3,000 outpatients who visited the diagnostic and treatment departments of our university hospital and compared the personality of patients with malignant tumors to the personality of patients with non-malignant tumors.

## Methods

Of 5,013 outpatients who visited the diagnostic and treatment departments of our university hospital between January 24 and January 28, 2011, 3,055 patients (collection rate, 60.9%) provided valid answers on the personality inventory. Of these, 3,003 patients aged 20 years or older were included in the analyses.

The personality inventory used was the Japanese version of the Maudsley Personality Inventory (MPI), which was first published in English by Jensen in 1958 [4]. MPI comprises 80 items assessing three personality fields: introversion/extroversion, neurotic tendencies, and lying tendencies. In this inventory, introversion/extroversion score (E-score) is evaluated on a 48-point scale, neurotic tendency score (N-score) on a 48-point scale, and lying tendencies score (L-score) on a 40-point scale. As shown in Table 1, a higher E-score indicates greater extroversion; a higher N-score indicates a greater neurotic tendency; and a higher L-score indicates a greater lying or exhibitionistic tendency [5,6]. This study was conducted after obtaining approval from the ethics committee of our university (approval no. 1143) and information regarding patient age, sex, and the department visited (multiple answers were allowed) were obtained while protecting patient anonymity. In our hospital, the disease name, such as cancer or malignant tumor, was in principle told to the patients themselves, and whether they were currently visiting our hospital due to a malignant tumor was answered by “Yes” or “No”.

**Table 1:** Judgment criteria of Japanese Maudsley personality inventory

**Score**	**Scale (point)**	**Judgment**
E score	0–9	extreme introversion
	10–20	slight introversion
	21–31	average
	32–41	slight extroversion
	42–48	extreme extroversion
N score	0–8	scarce neurotic tendency
	9–18	low neurotic tendency
	19–28	average
	29–38	neurotic tendency
	39–48	high neurotic tendency
L score	0–25	normal
	26–40	frequent lying and exhibitionistic tendency

Of the 3,003 patients investigated in this study, 1,193 were men and 1,810 were women, and the mean age was 63.1 ± 9.5 years. There were 603 patients in the malignant tumor group (M group) and 2,400 patients in the non-malignant tumor group (non-M group). The mean age of the M group was 65.3 ± 12.3 years and that of the non-M group was 62.4 ± 10.5 years; thus, the M group was slightly older, but there was no significant difference between the groups. With regard to sex, the M group included 255 men and 348 women, whereas the non-M group included 938 men and 1,462 women, showing no significant sex differences between the groups. The numbers of patients in both groups who visited each department (multiple answers allowed) are shown in Table 2. Because our hospital is a university hospital that conducts advanced treatments, fewer patients with end-stage cancer received palliative care alone and more patients with cancer were being treated aggressively.

**Table 2:** Number of patients presenting to each department

**Hospital department**	**Number of patients**	
	**M-group**	**Non-M-group**
Family medicine	2	60
Cardiology	8	129
Oncology	35	49
Diabetes, metabolism, and endocrinology	16	120
Neurology	6	80
Nephrology	0	42
Haematology	28	50
Gastroenterology and hepatology	44	137
Respiratory medicine	18	112
Gastrointestinal surgery	22	64
Hepatobiliary pancreatic surgery	14	56
Cardiovascular surgery	2	64
Neurosurgery	6	86
Oral and maxillofacial surgery	24	160
Thoracic surgery	14	20
Breast surgery	82	90
Orthopaedic surgery	44	184
Otorhinolaryngology	32	146
Nephro-urologic surgery	48	102
Obstetrics and gynaecology	78	145
Ophthalmology	14	260
Dermatology	30	162
Radiology	31	29
Pain clinic	4	26
Others	1	27

The checkpoints were E-, N-, and L-scores, which were compared between the M group and non-M group. Patient age was classified into three age groups, 20–49 years, 50–69 years, and 70 years or older, and the E-, N-, and L-scores of the M group and non-M group in each age group were compared. The patients were also divided into two groups by sex and the E-, N-, and L-scores in the M group and non-M group were also compared between men and women. Student’s t-test was used for statistical analysis and p < 0.05 was considered to indicate a significant difference.

## Results

Average E-score was 27.5 ± 11.6 points in the M group and 26.6 ± 11.8 points in the non-M group, indicating that patients in the M group were slightly more extroverted than those in the non-M group, although this difference was not significant. Average N-score was 16.8 ± 11.5 points in the M group and 17.7 ± 12.2 points the non-M group, indicating that patients in the M group had a slightly lower neurotic tendency than those in the non-M group; however, again, this difference was not significant. Average L-score was 17.6 ± 7.0 points in the M group, significantly higher than that in the non-M group (16.8 ± 7.4 points; p < 0.01), suggesting that more of the patients in the M group had lying or exhibitionistic tendencies. Investigation by age and sex revealed no significant differences in E-, N-, and L-scores between the M group and the non-M group (Tables 3 and 4).

**Table 3:** **Age of patients and scores in malignant group (M group) and non-malignant group (non-M group**)

**Age of patients (years)**	**M group (n = 603)**			**Non-M group (n = 2400)**		
	**E-score**	**N-score**	**L-score**	**E-score**	**N-score**	**L-score**
20–49	27.3 ± 13.1	17.5 ± 11.6	17.7 ± 6.4	26.5 ± 12.1	18.4 ± 13.0	16.4 ± 6.6
50–69	27.1 ± 11.0	16.5 ± 11.2	17.1 ± 5.8	26.3 ± 12.4	17.4 ± 12.4	16.4 ± 6.8
70-	28.0 ± 11.7	16.3 ± 11.5	18.0 ± 7.0	27.1 ± 11.3	17.2 ± 11.3	17.7 ± 6.9

No significant differences between the two groups.

**Table 4:** **Gender of patients and scores in malignant group (M group) and non-malignant group (non-M group**)

**Gender**	**M group (n = 603)**			**Non-M group (n = 2400)**		
	**E-score**	**N-score**	**L-score**	**E-score**	**N-score**	**L-score**
Male	27.4 ± 11.9	16.5 ± 12.1	17.4 ± 6.9	26.9 ± 11.9	17.3 ± 11.0	16.7 ± 7.1
Female	27.8 ± 11.2	17.0 ± 12.2	17.8 ± 7.3	26.1 ± 11.8	18.0 ± 12.2	17.0 ± 7.4

No significant differences between the two groups.

## Discussion

The possibility that personality may be a risk factor for the development of malignant tumors has been suggested [2,7,8]. Eysenck *et al*. [2] mentioned that personality, rather than smoking, may be strongly associated with the development of lung cancer. Temoshok *et al*. [8], reported that persons who tend to inhibit feelings such as anger or fear and make an effort to give a healthy impression may be more likely to develop malignant melanoma. However, the results of a few large cohort prospective study revealed that personality has no association with the development of cancer [9-11]. It had also been reported that personality may influence the progression or mortality of malignant tumors via action on the immune or endocrine systems [12]. However, the results of recent prospective studies by Nakaya *et al*. suggest that psychological elements do not significantly influence the progression or mortality of cancer [13,14].

Although personality is considered to have little influence on the development, progression, or mortality of malignant tumors, personality is strongly associated with the quality of life of patients [15]. Thus, it is very important for health professionals to assess the personality of patients with malignant tumors. Because few studies have investigated the personality of patients with malignant tumors, the data from the present study are expected to be of value.

With regard to the extroversion/introversion and neurotic tendencies of patients with malignant tumors, Kisen *et al.*[16] reported that male patients with lung cancer tended to be extroverted with a low neurotic tendency. Furthermore, Coppen *et al*. [17] found that patients with cancer were more extroverted and less neurotic than patients who did not have cancer. The results of the present study showed that the personality of patients with malignant tumors was slightly more extroverted and less neurotic than patients with non-malignant tumors. Based on these results, we consider that this may reflect the desire of patients with malignant tumors to establish positive relationships with the people around them and to lead an ordinary life without fear and worry in order to suppress the fear of their illness and death.

There have been very few studies on the evaluation of lying tendencies (L-score) in patients with malignant tumors. Hahn *et al*. [18] reported that the L-score was high in patients with breast cancer. The results of the present study revealed that L-score was significantly higher in the M group than in the non-M group. It has been reported that persons with higher L-scores on MPI tend to maintain an ideal self image and express themselves through exhibitionism [19]. Based on the above, we suggest that patients with malignant tumors are motivated to present themselves as healthy to others and to adopt a mindset of conquering their cancer and seeking a quality of life higher than that imagined by their doctors.

The limitations of the present study include the fact that data were obtained using only MPI from just one investigation at a single facility, the short study period, the inclusion of relatively few patients with end-stage cancer, and the lack of investigation regarding cancer type or disease name, permorbid characters, past histories and educational levels in all subjects**.** Therefore, we plan to conduct further detailed investigations using personality inventories other than MPI and in other facilities, such as hospices.

## Conclusion

Outpatients with malignant tumors showed a significantly higher L-score on MPI when compared with patients with non-malignant tumors. One reason for this may be that patients with malignant tumors are motivated to present an outward image of themselves as healthy and able to conquer their cancer. These results stress the importance of taking the mentality of patients with cancer into consideration when conducting treatment and care.

## Disclosure of authors

The manuscript submitted does not contain information about medical device(s)/drug(s). No funds were received in support of this work. No benefits in any form have been or will be received from a commercial party related directly to the subject of this manuscript. We certify that all applicable institutional and governmental regulations concerning the ethical use of human volunteers were followed during the course of this study.

## Competing interests

The authors declare that they have no competing interests.

## Authors’ contributions

All authors read and approved the final manuscript.
